# Early Versus Delayed Repair of Proximal Hamstring Tendon Ruptures: A Systematic Review and Meta-analysis

**DOI:** 10.1177/03635465261456228

**Published:** 2026-06-26

**Authors:** Muzammil Akhtar, Marlena Ramanis, Kory Pasko, Dean Wang

**Affiliations:** * Department of Orthopaedic Surgery, Saint Joseph's Medical Center, Stockton, California, USA; † University of California Irvine School of Medicine, Irvine, California, USA; ‡ Department of Orthopaedic Surgery, University of California Irvine, Irvine, California, USA; Investigation performed at the Department of Orthopaedic Surgery, University of California Irvine, Irvine, California, USA

**Keywords:** groin pain, hip/pelvis/thigh, muscle injuries, tendinosis

## Abstract

**Background:**

Early recognition and repair of proximal hamstring tendon ruptures is critical, as delays can complicate surgical repair. However, the influence of surgical timing on patient outcomes remains unclear, with previous studies reporting variable results.

**Purpose:**

To evaluate the effect of early versus delayed repair on clinical outcomes after primary proximal hamstring tendon repair.

**Study Design:**

Meta-analysis.

**Methods:**

A search was performed in accordance with the PRISMA (Preferred Reporting Items for Systematic Reviews and Meta-Analyses) guidelines in the PubMed, Embase, and Scopus databases. Studies were included if they (1) reported outcomes of primary proximal hamstring tendon repair, (2) had ≥12 months of follow-up, (3) included patients ≥18 years, and (4) compared early versus delayed treatment. Studies were excluded if they examined outcomes of proximal hamstring tendon repair/reconstruction using an allograft. Early repair was defined as surgery within 4 to 6 weeks of injury. Risk ratios (RRs) were calculated using a random-effects model, with significance set at *P* < .05.

**Results:**

Twelve studies were included, encompassing 992 early and 728 delayed repairs. The mean patient age ranged from 29.7 to 53.8 years in the early group and from 29.2 to 51.8 years in the delayed group. The mean follow-up ranged from 24 to 58.8 months. Nine studies reported patient-reported outcomes (PROs) at the latest follow-up; 3 demonstrated ≥1 significantly higher PRO scores in the early repair group. Return-to-sport/activity was reported in 5 studies, but was heterogeneous: 1 study reported a significantly higher rate of sports cessation after delayed repair, whereas others showed no difference in return-to-sport metrics. Early repair was associated with a significantly reduced risk of postoperative sciatica (RR, 0.26; *P* = .009) and retear (RR, 0.51; *P* = .03). Risk of peri-incisional numbness (RR, 0.48; *P* = .37), infection (RR, 0.51; *P* = .43), and revision (RR, 0.63; *P* = .36) were not significantly different between early and delayed repair groups.

**Conclusion:**

Patients undergoing proximal hamstring tendon repair within 4 to 6 weeks of injury can expect mostly similar PROs and a significantly lower risk of retear and sciatica compared with patients undergoing repair after 4 to 6 weeks of injury.

Proximal hamstring injuries encompass a broad spectrum of severity, ranging from mild tendinosis and low-grade strains to complete 3-tendon ruptures.^14^ The most common indications for surgical repair are complete 3-tendon ruptures, 2-tendon ruptures with >2 cm of retraction, and partial tears refractory to conservative management.^2,10^ The diagnosis can often be delayed, particularly in patients with a history of recurrent strains or chronic tendinopathy who may be referred for continued nonoperative treatment.^16,32^ Additionally, diagnosis is often delayed due to insurance barriers for magnetic resonance imaging (MRI) authorization.

Timely recognition of proximal hamstring tendon ruptures is essential to enable timely surgical repair, as a longer interval from injury to surgery can lead to increased tendon retraction, scarring, muscle atrophy, and tethering of the sciatic nerve, all of which can complicate surgical repair.^16,19,28^ However, the impact of injury chronicity on the clinical outcomes of proximal hamstring tendon repair remains unclear. While some studies suggest superior outcomes with early repair, others report no significant difference between early and delayed repair.

The purpose of this systematic review was to compare the clinical outcomes of early versus delayed proximal hamstring tendon repair in patients with proximal hamstring tendon injuries. We hypothesized that patients treated with early repair of acute injuries, defined as surgery performed within 4 to 6 weeks of injury, would demonstrate improved outcomes compared with those treated with delayed repair of chronic injuries. Outcomes of interest included patient-reported outcomes (PROs), return to sport (RTS)/activity, rerupture rates, and need for revision surgery.

## Methods

### Search Strategy

A search following the PRISMA (Preferred Reporting Items for Systematic Reviews and Meta-analyses) guidelines was conducted on September 1, 2025, in the PubMed, Embase, and Scopus databases. The following Boolean search phrase was used: (proximal hamstring AND (repair OR refixation)). Studies were included if they (1) reported outcomes of primary proximal hamstring tendon repair, (2) had a minimum follow-up of 12 months, (3) had a mean patient age of >18 years, and (4) compared clinical outcomes for patients with early versus delayed repair. The cutoff for early versus delayed repair was defined as 4 to 6 weeks from the initial proximal hamstring tendon injury.^1,8^ The exclusion criteria included studies that defined early versus delayed intervention differently, studies reporting outcomes of proximal hamstring tendon repair or allograft reconstruction, case reports, review articles, technique papers, animal studies, non-English articles, and expert opinions.

### Study Selection

Two independent reviewers (M.A, K.P.) assessed studies against the eligibility criteria from the initial database search. If they were not unanimous in their decision, a third reviewer (D.W.) was consulted to determine whether to include or exclude the study. All included articles underwent a rigorous search of their reference lists to identify additional studies that met our inclusion criteria and could be added to the systematic review.

### Data Extraction

Study variables extracted from the studies included authors, level of evidence (LOE), journal, study design, publication year, study period, patient inclusion and exclusion criteria, definition of early versus delayed repair, surgical technique, patient characteristics, time from injury to surgery, tear characteristics, follow-up time, PROs, activity or sports involvement, muscle strength, and adverse postoperative events. All extracted data were compiled for analysis using Microsoft Word (Microsoft Office 2011; Microsoft).

### Quality Assessment

Methodological quality for all studies was assessed using the methodological index for non-randomized studies (MINORS) score, which is reported on a scale of 0 to 24 for comparative studies. Two authors (M.A., K.P.) scored each article in the systematic review. Each author scored the articles individually before the authors reviewed their scores, and any discrepancies were resolved by re-reviewing the articles until a consensus was reached.

### Statistical Analysis

Descriptive statistics, such as means, percentages, standard deviations, ranges, medians, and interquartile ranges, are reported in this review when applicable and available from the individual studies. *P* values are also reported, when available, to indicate whether a given variable differed significantly between the early and delayed groups. *P* < .05 was considered statistically significant. The risk ratio (RR), 95% CI, and *P* value of complications were calculated. The meta-analysis was performed using a random-effects model. Heterogeneity was determined using the *I*^2^ statistic. Forest plots were generated using Review Manager (Version 5.4.1; Cochrane).

When calculating the RR, if the complication events were 0 in both the early and delayed groups in a single study, this would result in a division by 0 error, preventing the calculation of the RR. If that were the case for any study in the meta-analysis, the Haldane-Anscombe correction was applied, adding 1 event and 1 patient to the total number of patients in each study, allowing for an approximation of the RR with high accuracy.

## Results

### Article Selection Process

After searching the PubMed, Embase, Scopus, and Scopus databases, 1038 studies were identified; 461 were duplicates and were removed. The titles and abstracts of the remaining 622 studies were screened against our inclusion and exclusion criteria, and 563 were deemed irrelevant. Next, the full text of the remaining 59 studies was evaluated, and of those, 47 studies were deemed irrelevant as they did not stratify outcomes based on chronicity of symptoms or defined early versus delayed treatment differently. The remaining 12 studies were included in this systematic review (Figure 1).

**Figure 1.**
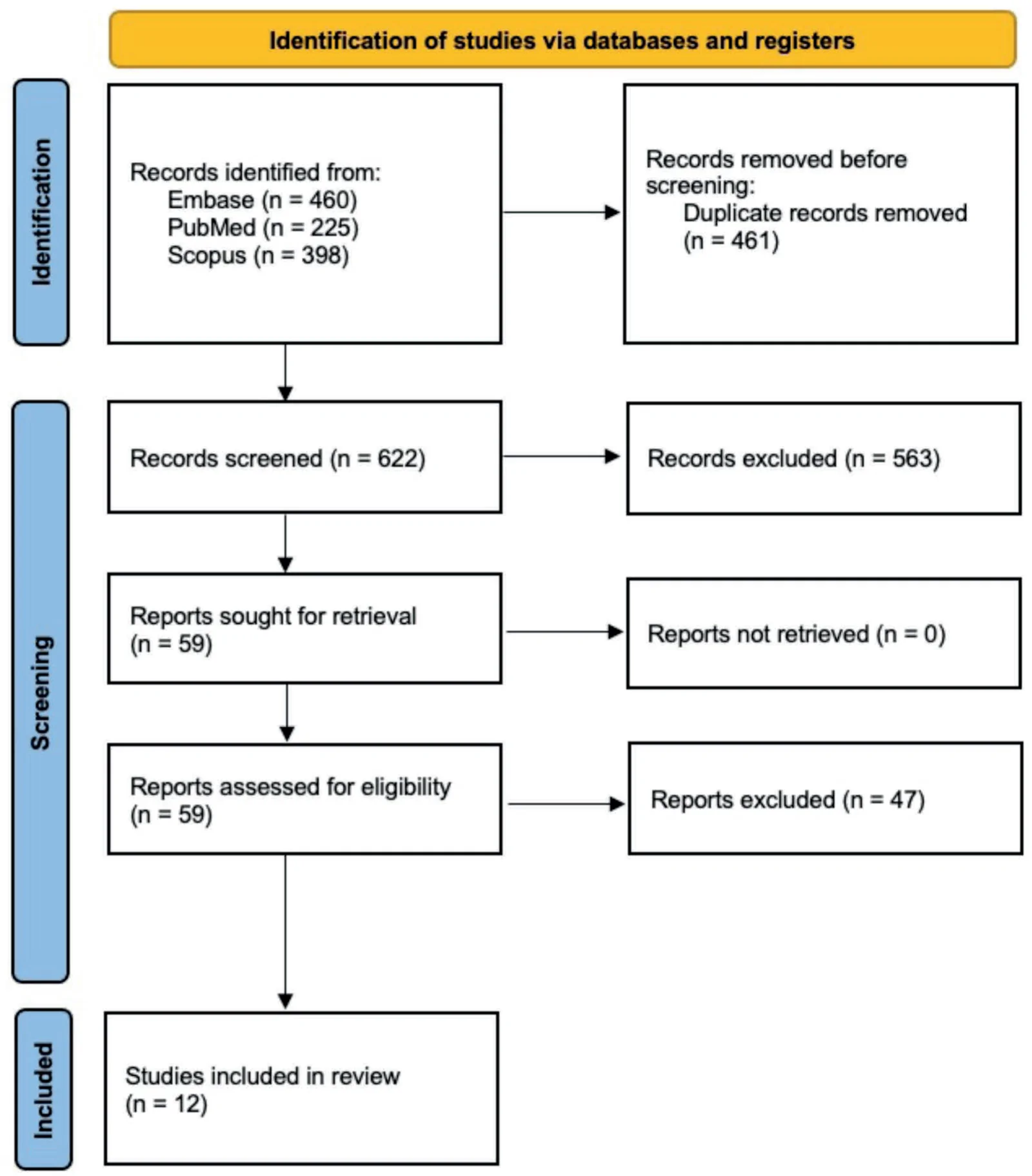
Flow chart depicting the article selection process.

### Study and Patient Characteristics

Twelve studies published between 2012 and 2024 were included. Six studies were LOE 3, and 6 were LOE 4. Ten studies had a retrospective study design, and 2 had a prospective study design. The mean ± standard deviation (range) of the MINORS score of all studies was 19 ± 2.1 (16-21). Eleven studies performed proximal hamstring repair using an open surgical technique, whereas 1 study included patients who underwent either open or endoscopic repair. Five studies set a cutoff of 4 weeks, and 6 studies set a cutoff of 6 weeks. One study evaluated outcomes in the same patient cohort, with cutoffs at both 4 and 6 weeks (Table 1).

**Table 1:** Study and Patient Characteristics*^*a*^*

Author	Study Design	MINORS	Surgical Technique	Early/ Delayed Cutoff, Weeks	Patients, M/F		Age, Years		Time to Surgery, Weeks		Follow-up, Months	
					Early	Delayed	Early	Delayed	Early	Delayed	Early	Delayed
Best et al^6^	Retrospective series	16	Open	4	143	31	48.9 ± 14.6		NR		26.2 ± 15.5	
Cohen et al^9^	Retrospective series	16	Open	4	40	12	47.7		NR		33 (12-76)	
Fenn et al^13^	Retrospective cohort	20	Open and endoscopic	4	27	48	56.5		NR		30.5 ± 5.1	
Lefèvre et al^20^	Retrospective case-control	20	Open	4	390	350	45.9 ± 13.6		NR		58.8 ± 46.8	
				6	427	313						
Léger-St-Jean et al^22^	Retrospective cohort	20	Open	4	22 (13/7)	16 (8/6)	47.1 ± 13.4	46.2 ± 19.9	1.9 (1.3-2.6)	18.7 (6-38.6)	49.2 ± 24	
Sandmann et al^27^	Retrospective cohort	16	Open	4	9	7	46.8		9.1 ± 10.4 (0.4-27.3)		55.9	
Barnett et al^4^	Retrospective series	19	Open	6	38	94	42.7	44.2	3.6	58.1	54.4	48.3
Kanakamedala et al^16^	Retrospective cohort	21	Open	6	19 (8/11)	19 (8/11)	52.6 ± 10.5	50.3 ± 9.4	1.6 (1.0-4.6)	16.9 (10.0-32.9)	62.4 ± 14.4	55.2 ± 22.8
Shambaugh et al^29^	Retrospective cohort	21	Open	6	73 (34/39)	20 (3/17)	51.2	48.2	2.7	89.6	43.6	39.5
Subbu et al^30^	Prospective series	22	Open	6	78 (51/27)	34 (25/9)	29.7	29.2	3.1	23.5	Min 24	
Willinger et al^33^	Retrospective series	17	Open	6	71 (41/30)	23 (12/11)	53.8 ± 11.7	50.7 ± 13.8	2.6 ± 1.5 (0.4-6.0)	23.6 ± 29 (6.1-138.6)	56± 25.9	56.9 ± 31.4
Wood et al^34^	Prospective series	20	Open	6	82 (45/37)	74 (29/45)	46.2	51.8	3.1 (0.3-5.7)	17.9 (6.1-5652.1)	44.4	45.6

a Age, time to surgery, and follow-up are reported as mean ± SD (range).

M/F, male/female; Min, minimum; MINORS, methodological index for non-randomized studies; NR, not reported.

In total, 992 patients underwent early surgical repair, and 728 patients underwent delayed repair. The mean age of patients treated with early repair group (reported in 7 studies) ranged from 29.7 to 53.8 years, compared with 29.2 to 51.8 years in the delayed group. The mean time to surgery (reported in 7 studies) ranged from 1.6 to 3.6 weeks for early repair and from 16.9 to 89.6 weeks for delayed repair.

### Preoperative Tear Characteristics

Details on partial versus complete tears were reported in 7 studies. Two studies included only patients with complete 3-tendon avulsions. In the remaining 5 studies, the proportion of partial tears ranged from 0% to 21.9% in the early repair group and from 31.2% to 60% in the delayed group. Among the 3 studies that directly compared groups, 2 reported a significantly higher incidence of partial tears in the delayed group.

The mean degree of tendon retraction, reported in 4 studies, ranged from 3.4 to 4.7 cm in early repair patients and from 2 to 4.6 cm in delayed repair patients, with all 4 studies reporting no significant difference between groups. One study reported on the proportion of patients with >3 cm retraction and found a significantly higher incidence in the early group (Table 2).

**Table 2:** Tear Characteristics*^*a*^*

Author	Partial Tears			Complete Tears			Tendon Retraction		
	Early, %	Delayed, %	*P*	Early, %	Delayed, %	*P*	Early, cm	Delayed, cm	*P*
Best et al^6^	0	0	**-**	100	100	**-**	NR		
Léger-St-Jean et al^22^	0	31.2	**.005**	100	68.8	**.005**	3.4 ± 3.4	2 ± 3.8 cm	>.05
Barnett et al^4^	5.3	36.2	NR	94.7	63.8	NR	NR		
Kanakamedala et al^16^	15.8	36.8	.28	84.2	63.2	.28	4.7 ± 3	3.5 ± 3	.2
Shambaugh et al^29^	21.9	60	**.002**	78.1	40	**.002**	56.2 with >3	35 with >3	**.042**
Subbu et al^30^	0	0	-	100	100	-	4	4.6	.4
Wood et al^34^	14.3	45.9	NR	85.7	54.1	NR	4.5	4	.466

a Tendon retraction is reported as mean ± SD or %. Bold *P* values indicate statistical significance. NR, not reported.

### Patient-Reported Outcomes

Nine studies reported on ≥1 PROs at a mean final follow-up ranging from 26.2 to 62.4 months. At the latest follow-up, early versus delayed repair group demonstrated the following range of PROs: Perth Hamstring Assessment Tool (PHAT) (76.9-95.8 vs 60.6-94.2), Lower Extremity Functional Scale (LEFS) (73.9-93.7 vs 71.5-79.8), Custom Marx Activity Rating Scale (MARS) (19.4-25 versus 18.7-25), MARS (5.5-10 vs 5-10.4), Tegner (4.7-6 vs 4-5.1), and visual analog scale (VAS) (0.7-2 vs 0.8-1.3).

Significant differences in at least 1 PRO score favoring early repair were reported in 3 studies. Cohen et al^9^ reported that the Custom MARS scores were significantly higher in the early treatment group. Léger-St-Jean et al^22^ reported that the LEFS, Single Assessment Numeric Evaluation, and Custom MARS scores were significantly higher in the early treatment group.^22^ Kanakamedala et al^16^ reported that the PHAT score was significantly higher in the early treatment group. All other PROs showed no significant differences between the early versus delayed groups, and no study found superior outcomes in the delayed group (Table 3).

**Table 3:** PROs and Sports/Activity Involvement*^*a*^*

Author	PROs				Sport/Activity Involvement			
	Outcome Measure	Early	Delayed	*P*	Measure	Early	Delayed	*P*
Best et al^6^	PHAT	81.3	71.4	.144	NR			
Cohen et al^9^	LEFS	76.2 (62-80)	71.5 (50-80)	.22	NR			
	MARS	10 (1-16)	10.4 (2-16)	.6				
	Custom LEFS	71.4 (48-80)	70.8 (47-80)	.72				
	Custom MARS	20 (20-20)	18.7 (12-20)	**.001**				
Léger-St-Jean et al^22^	LEFS	93.7 ± 11.1	79.8 ± 28.8	**.046**	Days to return to activity	120 (60-240)	180 (90-210)	>.05
	SANE	91.3 ± 8.2	83.8 ± 14.3	**.047**				
	MARS	5.5 (4-8)	5 (4-8)	>.05				
	Custom MARS	25 (25-25)	25 (22.5-25)	**.0087**				
Sandmann et al^27^	Lysholm	90.2 ± 3.4	93 ± 2.9	.1	No. of sports involved	3 ± 0.7	3.6 ± 1.3	.58
	WOMAC	96.3 ± 2.4	98.1 ± 2.7	.63	Training frequency, sessions per week	2.6 ± 0.7	2.1 ± 1.1	.37
	Tegner	4.7 ± 0.8	5.1 ± 0.7	.28	Training duration, minutes per week	129 ± 64	157 ± 56	.37
	UCLA	8.1 ± 0.9	8.1 ± 0.6	.96				
	VAS	0.7 ± 0.9	0.8 ± 0.6	.79				
Barnett et al^4^	Good to excellent	96%	92%	>.05	NR			
Kanakamedala et al^16^	PHAT	76.9 ± 18.8	60.6 ± 18.2	**.01**	NR			
	mHHS	79.5 ± 13.4	70.8 ± 18.5	.11				
	VAS	2.0 ± 2.6	1.3 ± 1.7	.35				
	Tegner	5 ± 2	4 ± 3	.31				
Shambaugh et al^29^	LEFS	73.9	73.4	.674	% with no functional limitations	60	60	>.999
	MARS	6.5	7.1	.710				
	Custom LEFS	645.5	67.9	.634				
	Custom MARS	19.4	19.5	.941				
	VAS	1	1.1	.770				
	UCLA	8.2	8.9	.061				
	Complete Satisfaction, %	90	90	>.999				
Subbu et al^30^	NR				RTS, %	98.7	91.2	NR
					Time to RTS, weeks	16 (12-32)	26.2 (18-41)	NR
Willinger et al^33^	Lysholm	85.7 ± 18.3	83.2 ± 15.9	>.05	RTS at preinjury level, %	50	47.6	**<.05**
	HHS	91 ± 12.6	90.6 ± 9.9	>.05	RTS at less than preinjury level, %	41.7	23.8	
	Tegner	6 (2-9)	5 (3-7)	>.05	Stopped sports, %	8.3	28.6	
	VAS	1.3 ± 1.8	2.2 ± 2.3	>.05				
Wood et al^34^	PHAT	95.8 ± 8.9	94.2 ± 9.2	.142	NR			
	SHORE	97.1 ± 11.4	95.7 ± 19.3	.132				

a PROs and sport/activity involvement are reported as mean ± standard deviation (range).

HHS, Harris Hip Score; LEFS, Lower Extremity Functional Scale; MARS, Marx Activity Rating Scale; mHHS, modified Harris Hip Score; NR, not reported; PHAT, Perth Hamstring Assessment Tool; RTS, return to sport; SANE, Single Assessment Numeric Evaluation; SHORE, Sydney Hamstring Origin Rupture Evaluation; UCLA, University of California, Los Angeles; VAS, visual analog scale; WOMAC, Western Ontario and McMaster Universities Osteoarthritis Index.

Two studies reported that a longer time to surgery was significantly correlated with worse PHAT scores (*r* = −0.228 and −0.47; *P* = .003 for both).^6,16^ Two additional studies stratified results by partial versus complete tears. Shambaugh et al^29^ found that patients with partial tears treated early had lower University of California, Los Angeles (UCLA) scores compared with those undergoing delayed treatment (8.1 vs 9.4; *P* = .014), whereas outcomes in complete tears were not significantly different. Wood et al^34^ found no difference in Sydney Hamstring Origin Rupture Evaluation (SHORE) scores between early and delayed repair for either partial or complete tears; however, the magnitude of SHORE score improvement was significantly greater in patients treated with early repair. Finally, Kanakamedala et al^16^ found that the degree of tendon retraction was not significantly different between the early versus delayed repair groups, and was not significantly correlated with the PHAT (*r* = 0.03; *P* = .87) or modified Harris Hip Score (mHHS) (*r* = −0.05; *P* = .77) across the cohort.^16^

### Activity or Sport Involvement

Five studies reported outcomes related to return to activity or sport (Table 3). Three studies found no significant differences in activity level, sports participation, or functional limitations between early- and delayed-repair groups.^22,27,29^ Willinger et al^33^ reported a significantly higher rate of inability to RTS in the delayed group (28.6% vs 8.3%) and a significantly higher rate of RTS at or above preinjury level in the early group.^33^ Subbu et al^30^ reported higher rates of RTS and a shorter time to RTS in the early repair group, although the study did not report statistical significance.

### Muscle Strength

Only 1 study formally assessed hamstring and quadriceps muscle strength and endurance. In that study, the mean preoperative hamstring strength of the involved limb was 24.2% ± 24.9%, and endurance was 36.8% ± 54.2% relative to the contralateral limb. At the latest follow-up, the mean hamstring strength improved to 81% ± 22% and endurance to 106.4% ± 49.6% relative to the contralateral limb. While specific values were not stratified by repair timing, no significant differences in follow-up strength or endurance were found between the early and delayed groups.^4^

Two additional studies assessed strength subjectively by patient self-report. Cohen et al^9^ found no significant difference in subjective strength of the operated leg versus contralateral leg between early and delayed repair groups (88.6% ± 13% vs 87.5% ± 13%; *P* = .77). Similarly, Shambaugh et al^29^ reported that near or full strength was perceived of the operated leg compared with the contralateral leg in a similar percentage of patients undergoing early versus delayed repair (79.5% vs 65%; *P* = .275, a finding consistent in both partial and complete tear subgroups.

### Adverse Postoperative Events

#### Sciatica

Five studies reported sciatica rates with a mean follow-up of 24 to 62 months. The pooled rate (range) in the early group was 6.5% (0%-36.8%), and in the delayed group, 24% (10.4%-52.6%). The risk of sciatica was significantly higher in the delayed group (RR, 0.26 [95% CI, 0.09-0.71]; *P* = .009; Figure 2A).

**Figure 2.**
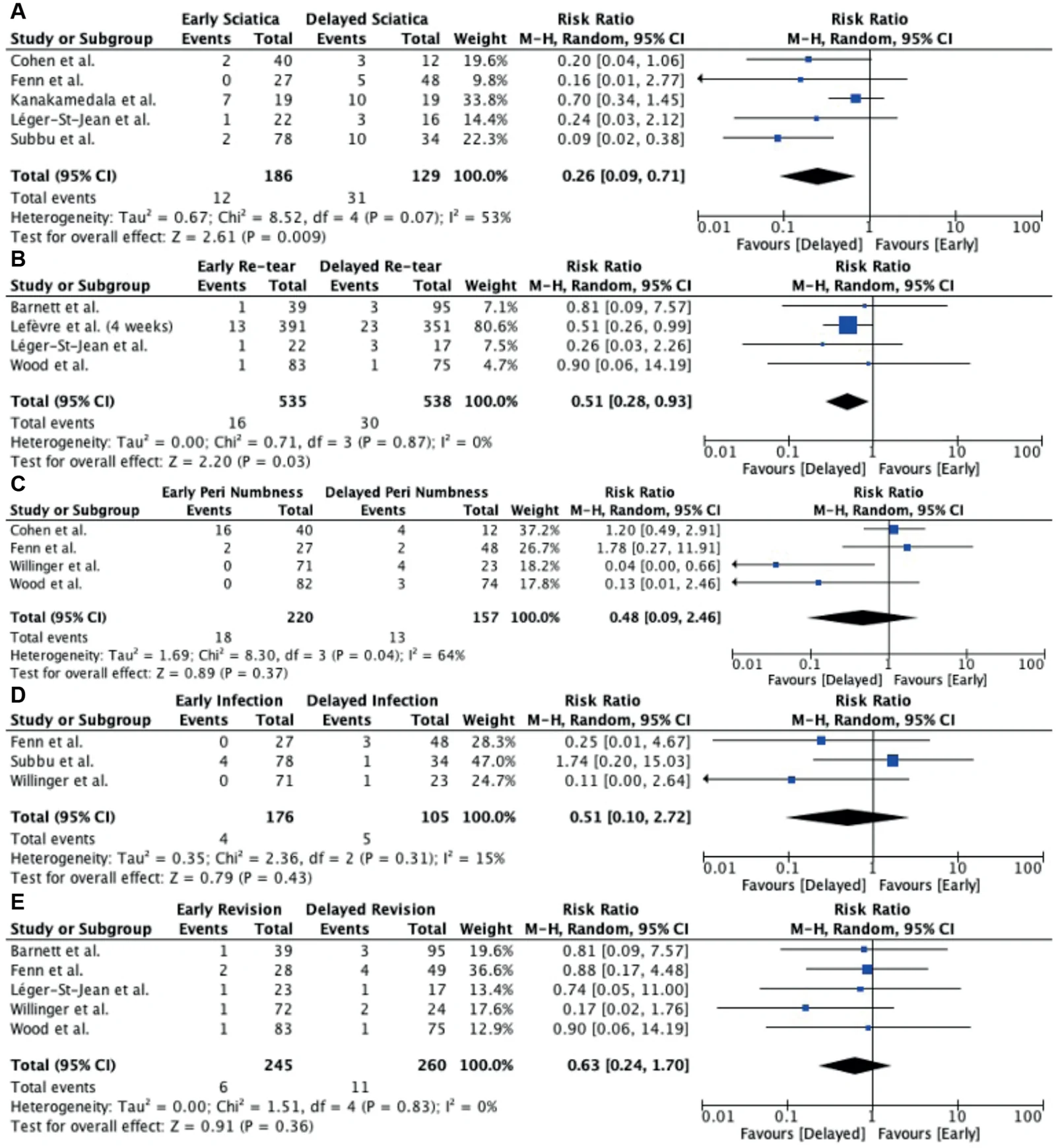
Forest plots demonstrating the risk of adverse postoperative events, (A) sciatica, (B) retear, (C) peri-incisional numbness, (D) infection, (E) revision, in early versus delayed groups. M-H, Mantel-Haenszel.

#### Retear

Four studies reported retear rates, with a mean follow-up of 44.6-58.8 months. The pooled rate (range) was 2.2% (0%-3.1%) in the early group and 4.9% (0%-6.3%) in the delayed group. The risk of retears was significantly higher in the delayed group (RR, 0.51 [95% CI, 0.28-0.93]; *P* = .03; Figure 2B).

#### Peri-incisional Numbness

Four studies reported peri-incisional site numbness rates, with a mean follow-up ranging from 30.5 to 56.9 months. The pooled rate (range) was 8.2% (0%-40%) in the early group and 8.3% (3%-33.3%) in the delayed group. The risk of peri-incisional site numbness was not significantly different between early and delayed groups (RR, 0.48 [95% CI, 0.09-2.46]; *P* = .37 (Figure 2C).

#### Infection

Three studies reported infection rates with a mean follow-up ranging from 24% to 56.9 months. The pooled rate (range) was 2.3% (0%-5.1%) in the early group and 4.8% (2.9%-6.3%) in the delayed group. The risk of infection was not significantly different between early and delayed groups (RR, 0.51 [95% CI, 0.10-2.72]; *P* = .43) (Figure 2D).

#### Revision

Five studies reported revision rates with a mean follow-up of 30.5-56.9 months. The pooled rate (range) in the early group was 0.4% (0%-3.7%), and in the delayed group, 2.4% (0%-6.3%). The risk of revision was not significantly different between early and delayed groups (RR, 0.63 [95% CI, 0.24 to 1.70]; *P* = .36; Figure 2E).

## Discussion

The primary findings of this systematic review were as follows: 3 of 9 studies reported at least 1 significantly better PRO score in the early repair groups. Three studies reported no significant differences in activity, sports involvement, or functional limitations at final follow-up between early- and delayed-repair groups. Patients treated with delayed repair demonstrated a significantly higher risk of sciatica and retear, with no significant differences in infection, peri-incisional numbness, or revision surgery.

A 2021 systematic review from Reza et al^26^ highlighted the need for standardized outcome measures in proximal hamstring tendon repair and recommended the use of RTS, isokinetic strength testing, LEFS, and PHAT. In the present systematic review, 5 studies assessed RTS/activity, but the outcome measures were heterogeneous; only 1 study reported a significantly higher RTS rate in the early repair group. Only 1 study completed an objective assessment of isokinetic strength testing and found no significant difference between groups. Furthermore, regarding PROs, the PHAT, SHORE, and Parisian Hamstring Avulsion Score (PHAS) are the only validated outcome measures designed for proximal hamstring tendon repair.^7,21,34^ In the present systematic review, three studies reported the PHAT score, with 1 favoring early repair and the other 2 studies reporting no differences. Two studies reported that a longer time to surgical repair was associated with worse PHAT scores. Wood et al^34^ was the only study to use the SHORE score, and they reported no significant difference in scores between groups. However, they reported that preoperative-to-follow-up improvement in the SHORE score was significantly greater in the early repair group. Lefevre et al^21^ recently validated the PHAS score, demonstrating a strong correlation with the Tegner and UCLA scores and reliable prognostic value for RTS.^21^ While the LEFS is not specific to proximal hamstring tendon repair, it has been shown to be strongly correlated with the PHAT.^25^ In this review, 3 studies reported the LEFS score, with 1 study favoring early repair. These findings underscore the need for future studies to adopt standardized, validated measures such as those proposed by Reza et al.^26^

Delayed repair increases surgical complexity due to increased fibrosis, adhesions, and tendon retraction, which heighten the risk of tethering of the sciatic nerve and its branches and postoperative neurologic complications, including foot drop, posterior thigh pain, and numbness or tingling.^1,15^ One study reported an 84.4% incidence of sciatic neuritis in patients with chronic tears.^11^ Increased scarring also complicates sciatic nerve mobilization, thereby increasing the rate of postoperative neurologic complications.^1,8^ In this review, 3 studies reported greater rates of postoperative sciatica, and 3 others reported neurologic symptoms in chronic injuries. Shambaugh et al^29^ additionally reported that patients with complete chronic tears had significantly longer operative times than those with acute tears (100 vs 64.7 minutes; *P* = .004), further highlighting the added technical challenge of repairing chronic injuries.^29^

With respect to tendon retraction, previous literature has suggested worse outcomes with greater tendon retraction in the setting of chronic injuries.^31^ However, 1 study in this review found no correlation between the degree of tendon retraction and PHAT or mHHS scores.^16^ The authors of that study suggested that after an initial period of retraction around the time of injury, retraction may not progress enough to impact clinical outcomes despite increasing complexity of the surgical repair.

While muscle atrophy in the setting of chronic proximal hamstring injuries has previously been thought to contribute to poor clinical outcomes after surgical repair, only 1 study has formally evaluated this association. Li et al^23^ used the Goutallier classification on preoperative MRI in patients with chronic injuries and found no association between muscle atrophy and time to surgery, tendon retraction, presence of a complete 3-tendon rupture, PHAT, or mHHS. Instead, increased age was significantly predictive of greater muscle atrophy (*P* = .005) and poorer mHHS (*P* = .037). Notably, all patients in that cohort only had mild to moderate atrophy (Goutallier grades 1-2). Another interesting finding by Li et al^23^ was that greater time to surgery was not significantly predictive of worse PHAT and mHHS, which may indicate that in chronic injuries, there may not be sufficient deterioration in tissue quality to affect clinical outcomes. Similarly, another study found that while chronicity was not significantly associated with worse outcomes, older age was a significant risk factor for revision surgery.^12^ Taken together, these findings suggest that patient age, rather than chronicity alone, may play a larger role in influencing outcomes.

Accurate and timely diagnosis is essential for identifying candidates for surgical repair. MRI remains the gold standard, yet access is often delayed due to insurance authorization requirements. Ultrasonography may serve as an accessible alternative when performed by experienced radiologists, although accuracy is operator-dependent and less consistent than MRI.^3,5, 17,18, 24^

### Limitations

This systematic review must be considered in the context of its limitations. Most included studies analyzed cohorts of complete and partial tendon tears, with most not differentiating between tear types when reporting outcomes. This may obscure outcome differences. Additionally, 3 studies did not specify surgical indications. While it can be implied that these studies utilized the generally recognized indications in the literature,^3,10^ different injury causes may act differently after surgical repair. For instance, a chronic, partial undersurface proximal hamstring tendon injury may heal differently from an acute, full-thickness tendon rupture, even if the surgical repair technique is the same. Furthermore, the definition of early versus delayed repair remains ill-defined, with nearly half of the studies using a 4-week cutoff and the other half using a 6-week cutoff. Lastly, the low LOE of the included studies and the heterogeneous use of outcome measures precluded pooling of data for meta-analysis.

## Conclusion

Patients undergoing proximal hamstring tendon repair within 4 to 6 weeks of injury can expect mostly similar PROs and a significantly lower risk of retear and sciatica compared with patients undergoing repair after 4 to 6 weeks of injury.
